# Rethinking cancer: current challenges and opportunities in cancer research

**DOI:** 10.1242/dmm.030007

**Published:** 2017-04-01

**Authors:** Ross Cagan, Pablo Meyer

**Affiliations:** 1 Icahn School of Medicine at Mount Sinai, Annenberg 25-40, Campus Box 1020, 1468 Madison Avenue, New York, NY 10029, USA; 2 Thomas J. Watson Computational Biology Center, IBM, Yorktown Heights, NY 10598, USA

## Abstract

Cancer therapeutics currently have the lowest clinical trial success rate of all major diseases. Partly as a result of the paucity of successful anti-cancer drugs, cancer will soon be the leading cause of mortality in developed countries. As a disease embedded in the fundamentals of our biology, cancer presents difficult challenges that would benefit from uniting experts from a broad cross-section of related and unrelated fields. Combining extant approaches with novel ones could help in tackling this challenging health problem, enabling the development of therapeutics to stop disease progression and prolong patient lives. This goal provided the inspiration for a recent workshop titled ‘Rethinking Cancer’, which brought together a group of cancer scientists who work in the academic and pharmaceutical sectors of Europe, America and Asia. In this Editorial, we discuss the main themes emerging from the workshop, with the aim of providing a snapshot of key challenges faced by the cancer research community today. We also outline potential strategies for addressing some of these challenges, from understanding the basic evolution of cancer and improving its early detection to streamlining the thorny process of moving promising drug targets into clinical trials.

## Abstract (precis)

**Summary:** Pablo Meyer and Ross Cagan discuss the main themes emerging from a recent workshop ‘Rethinking Cancer’, highlighting the key challenges faced by the research community and outlining potential strategies to promote translation of basic findings.

In November 2016, a unique workshop titled ‘Rethinking Cancer’ was hosted by The Company of Biologists, the not-for-profit publisher of Disease Models & Mechanisms. This workshop was structured as an ‘unconference’, during which formal presentations were replaced by question-based roundtable discussions. The discussions between the ∼35 attendees of this workshop – the majority were academics with a few scientists from industry – mainly gravitated towards why model systems can be cured of cancer, but people often cannot. One overarching conclusion is that the true focus of cancer biology must continue to move from the discovery of essential biological mechanisms needed to understand cancer to the translation of these results to the clinic. The message is not to abandon the drive to understand the fundamental biology of cancer but to maintain a focus on therapeutics throughout the discovery process. Historically, research has been largely geared towards identifying the drivers of tumor progression; however, therapeutically targeting these drivers can lead to unacceptable toxicity due to their roles in overall body homeostasis. Thus, it is important to rethink how we approach the complex problem of curing cancer to obtain the much-heralded impact on patients. Bringing together academic and industry researchers for a free-wheeling, two day discussion proved a good way to identify points where we can better focus to increase the impact of our work.

This Editorial summarizes the outcome of the workshop, with a particular focus on challenges such as: (1) obtaining a better understanding of what clinical trials entail and possibilities for re-designing them; (2) improving initial detection of cancer in patients; (3) improving access to resources for researchers; (4) developing more predictive preclinical models of cancer; and (5) using genomics and systems biology more effectively.

## Understanding what clinical trials entail

An important recommendation from this workshop is that basic researchers, institutions and funding agencies should take more responsibility to learn and encourage how to move from bench to bedside to allow better outcomes of drug discovery projects. This involves learning the full path to getting candidate therapies into trials, including becoming informed on intellectual property and compliance (Cagan, 2016). Scientists should take the opportunity to connect better with clinicians to understand the full palate of issues when treating patients, including financial considerations, difficulties in patient recruitment, potential for toxicity and difficulty of compliance with complicated dosing regimens. Training in the art of linking researchers and clinicians should begin early in graduate school to promote two-way interactions, with the aim of getting clinicians into the lab and students into clinics.

When moving promising drug hits towards the clinic, it is essential to understand pharmaceutical concepts, such as on- and off-target physiological effects, tumor shrinkage, pharmacokinetics and pharmacodynamics (PK/PD), therapeutic window (defined by the balance of efficacy and toxicity) and maximum tolerated dose (MTD; the highest drug dose that does not cause unacceptable side effects). Researchers should focus on discovery platforms that identify toxic effects early in the discovery phase. Engaging chemists early in the discovery process should enable focus on leads that are likely to show ‘druggable’ properties. Other points to consider when designing a screen or choosing a lead to follow include whether the proposed target is druggable when considering the whole body beyond the tumor. For example, while most cancer deaths are due to metastasis, anti-metastatic drugs are often considered a poor avenue forward due to the high cost and long time required for clinical trials. Key questions to ask when planning clinical trials include: Is it possible to identify a patient cohort? How much will the trials cost?

Whether clinical trials succeed or fail, we need to routinely analyze them for lessons learned. Related to this, more effort should be put into learning how classic drugs work. A notable example is provided by tamoxifen, which, despite being widely used and strongly effective, is poorly understood in terms of the mechanism of action. Yet it is cytostatic *in vitro* and leads to strongly improved overall survival in the adjuvant setting (International Breast Cancer Study Group, 1997). Similarly, we do not understand why platinum is curative for testicular germ cell cancer and (initially) effective in treating lung cancers, yet other tumors are resistant (Martin et al., 2008). These drugs set a high bar, and we should strive to understand their mechanism of action more fully.

**Figure:**
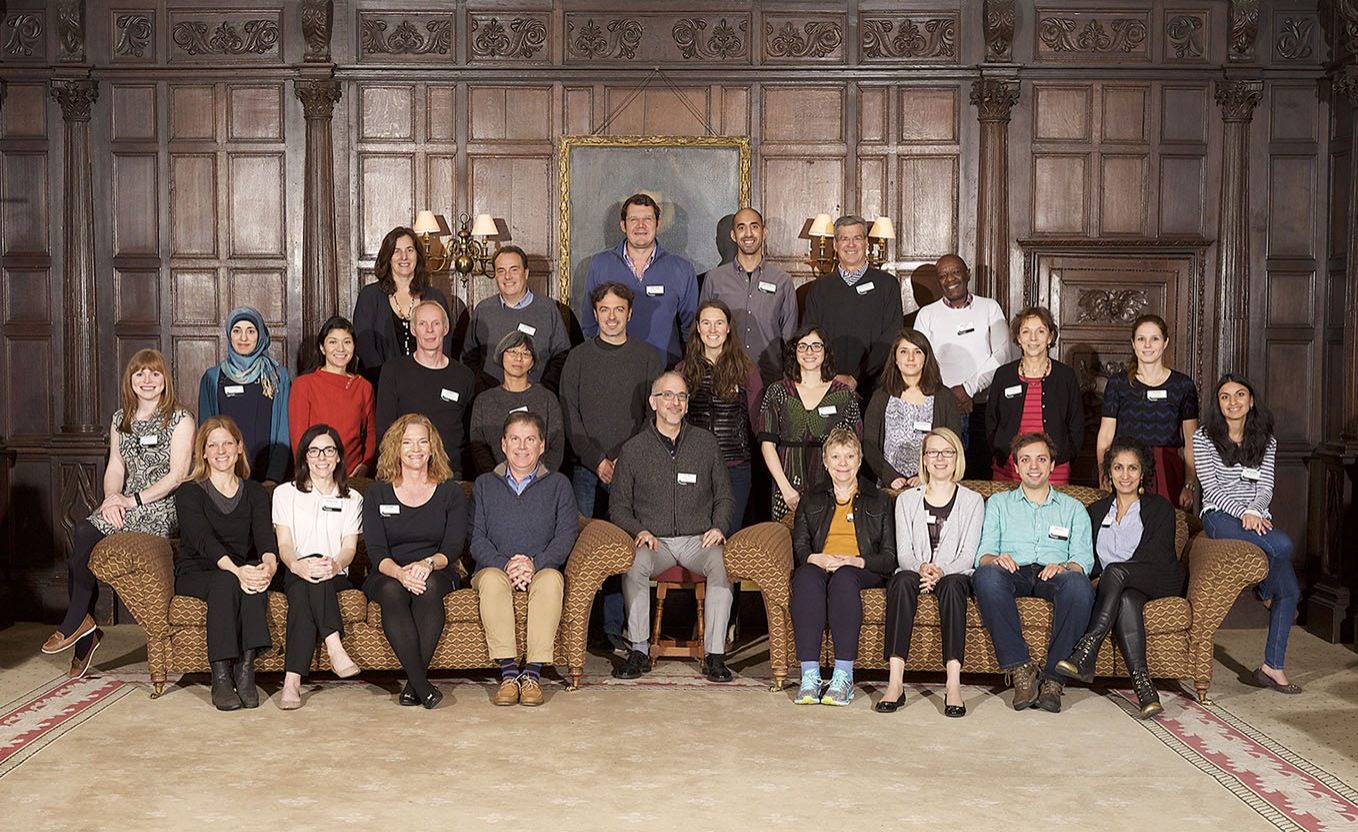
Delegates at The Company of Biologists ‘Rethinking Cancer’ workshop held at Wiston House, Steyning, UK on 20-23 November 2016. For further information, see http://www.biologists.com/workshops/rethinking-cancer-november-2016/.

## Challenges in improving initial detection

For most human diseases, prevention is generally preferable to finding a cure, both to improve the chances of successful treatment and as the less expensive approach. In cancer, early tumor detection with non-invasive imaging such as CT, MRI and PET scans – known as radiomics – is proving increasingly promising for discriminating between indolent and aggressive tumors (Frangioni, 2008). Surgery in particular can become especially effective, even curative. To guide surgeons, improved imaging holds the promise of better defining and removing tumor margins, and image recognition software can now classify skin melanomas (Esteva et al., 2017). However, these are expensive interventions that also increase an individual's exposure to radiation and can have a high false-positive rate.

The glucose analog ^18^F-fludeoxyglucose, used for PET imaging, measures the glycolytic flux of cancer cells based on their characteristic increased glucose uptake. This, in conjunction with other tracers, permits the detection of metabolic changes before tissue-level alterations occur (Challapalli and Aboagye, 2016). Further opportunity should be taken to exploit these resources to alleviate the need for biomarkers when assessing candidate therapeutic targets. Study of the clonal dynamics of tumor initiation [for example, in genetically engineered mouse models (GEMMs)] using technologies such as data analytics and exome sequencing could also facilitate early diagnosis. Promising anti-cancer approaches, such as the emerging field of immunotherapies, may prove more effective during earlier stages of the disease, before a patient's immune system is battered by advanced disease and by prior therapeutics. A key challenge is financial: the current medical insurance system in the US at least provides no financial support for refining prevention strategies such as cancer-preventing vaccines, a strategy that has proven successful for HPV. In general, approaching cancer as a community challenge can yield important benefits and is important for basic researchers to consider in their cancer studies.

## Better access to resources

The future of cancer research will increasingly rely on improved collaboration as we develop more sophisticated therapeutic, diagnostic and prognostic tools. Improving public availability of data will require agreement on specific standards for data handling, processing and sharing (Mardis, 2016). The DREAM mammography imaging challenge (http://sagebase.org/press-releases/the-digital-mammography-dream-challenge/) and several drug recombination challenges are a good example of the power of crowdsourcing using publicly available data (Saez-Rodriguez et al. 2016). The challenges we are facing are complex and will require an increasingly broad spectrum of expertise. From basic research to clinical trials and disease prevention, the cancer field needs to promote increased open access to resources such as gene expression and radiomics data, and patient tissue samples.

In addition, in light of rapid improvements in the development of organoids, *ex vivo* culture models and patient-derived xenograft models, new approaches to preserving tissue, such as cryo-preservation, would allow future work on cultured tumors/models and thus more comprehensive analysis. By standardizing cryo-preservation, tissues can be ‘resuscitated’ and studied, moving cancer pathology from purely structural analyses to functional studies that could positively impact patients as well as research.

## Developing better models

One ongoing question during this workshop was how to incorporate model organisms into the drug development process. The starting point of a new cancer study should be grounded in data from human patients or from epidemiological data; animal or cellular models should be designed to capture as much tumor complexity as possible. For example, tumors are much more than DNA mutations; finding pathway-based connections, such as between the oncogenes Ras and AKT (Hirabayashi et al., 2013) helps capture important aspects of cancer biology. Candidate therapeutics should be tested in multiple models, because the genetic background can be critical in explaining the effect of a particular mutation. Nonetheless, mouse models represent the key mammalian model used prior to moving drug hits into human trials.

A key point that emerged from the workshop is the importance of properly interpreting data from model systems: we should not expect patients to respond more robustly than our models do. Criteria that are used for patients should be used when testing therapies in mice; for example, mouse xenograft data should be displayed as waterfall plots, and at least 30% tumor shrinkage should be observed in the majority. This is the standard response evaluation criteria in solid tumors (RECIST) in clinical trials (Therasse et al., 2000), and humans will rarely outperform mouse models.

We also need a deeper understanding of epithelial dynamics to explain, for example, how surrounding cells and tissues influence tumor progression. By some criteria, cancer is a wound that never heals and parallels between tissue repair and cancer have been made (Dvorak, 1986). One exception is the case of pediatric cancers, where a single genomic alteration is the primary driver of the tumor and so expression of the mutation in a zebrafish model adequately phenocopies the disease (Eden et al., 2015). For the majority of cancers, we need better and more affordable pre-clinical ‘humanized’ models that model cancer pathways in a realistic biological context. One solution may derive from the opportunities afforded by gene-editing technologies such as CRISPR/Cas9. Ideally it would be possible to develop an animal model for every subtype of cancer, capturing more of the genomic complexity observed in patients. Organoid culture systems can often achieve this complexity; they provide a powerful tool to explore human cancer biology and a platform for disease modeling and personalized medicine. Examples using this rationale are primary tissues grown in culture (endodermal, pancreas, liver) and organoids grown in a Matrigel (with WNT) using the MCF10A cell line (Matsumoto et al., 2014).

Such approaches could help explain distinctive molecular features that depend on the type and origin of the cancer. For example, K-Ras is mutated in 83% of pancreatic cancers, but in breast and colon cancer Ras mutants typically do not develop into cancer (Vogelstein and Kinzler, 2014). The microenvironment of tumors could hold the key to explaining this heterogeneity. This environment, including the local microenvironment – where cancer cells co-exist with normal epithelium and stroma – is poorly understood at present. The metabolic context in some cases makes the tumor more aggressive, as shown by a Ras-Src mutant grown in sugar in thyroid cancer models (Mulligan, 2014; Hirabayashi, 2013).

Super-responders and non-responders to cancer drugs (Prasad and Vandross, 2015) are of increasing interest in the cancer community, warranting deeper exploration. For example, exploring the molecular basis of a ‘super-response’ could provide a strategy to find synthetic lethal genes. Two genes are synthetic lethal if one mutation leads to a viable cell but both mutations lead to death; for example, PARP inhibitors have been shown to be synthetic lethal in the context of a *BRAC2* mutation (Lord and Ashworth, 2017). Some patients become super-responders by matching a targeted therapy with a synthetic lethal partner (Kaelin, 2005).

More broadly, we poorly understand why different tumor types with similar gene drivers nonetheless respond differently to therapeutics. For example, chemotherapy works remarkably well for a subset of cancers such as lung (Einhorn, 2008), but the reason for this high sensitivity is unknown. A putative solution could come from better cancer monitoring through the development of multiplexed, multiparametric approaches instead of the use of single parameters, as this can help in identifying mechanisms for unexpected resistance to treatment (Prahallad et al., 2012).

## Opportunities from genomics and systems biology

A rapid advancement in genomics technologies combined with the low cost of sequencing have led to the generation of vast amounts of cancer genomic data in recent years. This ever-expanding wealth of data is needed in order to answer some of the most pressing new questions in cancer. What is the evidence that identifying more cancer risk mutations is of therapeutic relevance? How does the developmental lineage of a cell type inform about cancer? How do different cancer states – subtypes classified based on differences in gene expression – alter cancer cell dynamics? How and why do cancers retain lineage identity and does the cell of origin matter? These are in addition to long-pursued questions, such as why some transcriptional programs are robust to mutations (Califano and Alvarez, 2017).

The analysis provided by genomics and systems biology approaches is crucial, in particular as it has become clear that cancer is not a disease of genes but of pathways, highlighting the importance of identifying key ‘functional outputs’. In particular, one has to be careful when assessing the contribution of cancer mutations, as cancer networks are complex and robust; further, even ‘passengers’ that do not confer proliferative advantage may alter patient response to therapeutics. Computational approaches can identify common drivers (Califano and Alvarez, 2017) of cancer progression and can recommend drugs to match the genomic data. However, the accuracy of this method has not always proved to be high enough to be useful. Also, cancers are not isolated units in space and time; knowledge of their tissue of origin and their evolution (e.g. disease progression) is essential. These aspects are rarely captured by computational modeling. Exome sequencing in conjunction with crowdsourcing (Ewing et al., 2015) could provide insights into cell fate behavior of physiologically normal tissues as clonal analysis – detected by 150× deep sequencing – can uncover the somatic mutations that drive the transitions to disease states (Crowley et al., 2013). Other features such as exosomes, circulating tumor cells (CTCs) and free circulating DNA may help early detection of metastatic cells.

As cancer research matures to achieve better therapies and ultimately cures, it should connect more with other disciplines. Genomics provides quantitative data that would benefit from input from disciplines as varied as mathematics, physics, bioengineering, ecology and evolutionary biology.

## Future opportunities and challenges

Discussions during this workshop consistently underlined the need for coordinated efforts from multiple fields and from both the basic and clinical spheres in order for cancer research to have more direct clinical impact. This includes better understanding the path to clinical trials, improving and encouraging access to clinical resources for researchers, developing more sophisticated models of cancer that include clinical rigor and further exploitation of genomics and systems biology approaches in conjunction with other technologies. The significant challenge posed by cancer demands swift and accessible innovations that can be rapidly tested in a clinical environment. Because the development of new technologies thrives in an interdisciplinary environment, the pressing challenges of cancer research require collaboration across academia and industry with minimal financial or intellectual barriers.
